# A garment that measures brain activity: proof of concept of an EEG sensor layer fully implemented with smart textiles

**DOI:** 10.3389/fnhum.2023.1135153

**Published:** 2023-05-26

**Authors:** Eduardo López-Larraz, Carlos Escolano, Almudena Robledo-Menéndez, Leyre Morlas, Alexandra Alda, Javier Minguez

**Affiliations:** Bitbrain, Zaragoza, Spain

**Keywords:** electroencephalography (EEG), textile EEG, Dry-EEG, wearable EEG, smart textiles, neurotechnology, brain-computer interface (BCI), EEG dataset

## Abstract

This paper presents the first garment capable of measuring brain activity with accuracy comparable to that of state-of-the art dry electroencephalogram (EEG) systems. The main innovation is an EEG sensor layer (i.e., the electrodes, the signal transmission, and the cap support) made entirely of threads, fabrics, and smart textiles, eliminating the need for metal or plastic materials. The garment is connected to a mobile EEG amplifier to complete the measurement system. As a first proof of concept, the new EEG system (Garment-EEG) was characterized with respect to a state-of-the-art Ag/AgCl dry-EEG system (Dry-EEG) over the forehead area of healthy participants in terms of: (1) skin-electrode impedance; (2) EEG activity; (3) artifacts; and (4) user ergonomics and comfort. The results show that the Garment-EEG system provides comparable recordings to Dry-EEG, but it is more susceptible to artifacts under adverse recording conditions due to poorer contact impedances. The textile-based sensor layer offers superior ergonomics and comfort compared to its metal-based counterpart. We provide the datasets recorded with Garment-EEG and Dry-EEG systems, making available the first open-access dataset of an EEG sensor layer built exclusively with textile materials. Achieving user acceptance is an obstacle in the field of neurotechnology. The introduction of EEG systems encapsulated in wearables has the potential to democratize neurotechnology and non-invasive brain-computer interfaces, as they are naturally accepted by people in their daily lives. Furthermore, supporting the EEG implementation in the textile industry may result in lower cost and less-polluting manufacturing processes compared to metal and plastic industries.

## Introduction

The electroencephalogram (EEG) is a non-invasive technique for measuring the electrical activity produced by the brain (Biasiucci et al., 2019). Some of its most relevant applications are for diagnosis of conditions such as epilepsy, ADHD or sleep disorders, among others, as a brain mapping tool for neuroscience, psychology and electrophysiology research, and for brain-computer/machine interface (BCI/BMI) applications (Niedermeyer and Lopes da Silva, 2005). One trend of innovation in the EEG field in the last 2 decades addresses the evolution of EEG technology to enable its use for real-world research and real-life applications (Niso et al., 2023). This has led to the development of wireless systems, their miniaturization, low-density sensor layouts, the use of new conductive materials to produce gel-less sensors, improved usability for non-experts, and cost reduction, among many other innovations (Casson et al., 2010; Millán et al., 2010; Casson, 2019). All these efforts are directed toward the improvement of user acceptance, while trying to preserve signal quality. Although there has been very relevant progress in this direction, reliable EEG technologies that are affordable and widely accepted by the general population are still missing.

**FIGURE 1:**
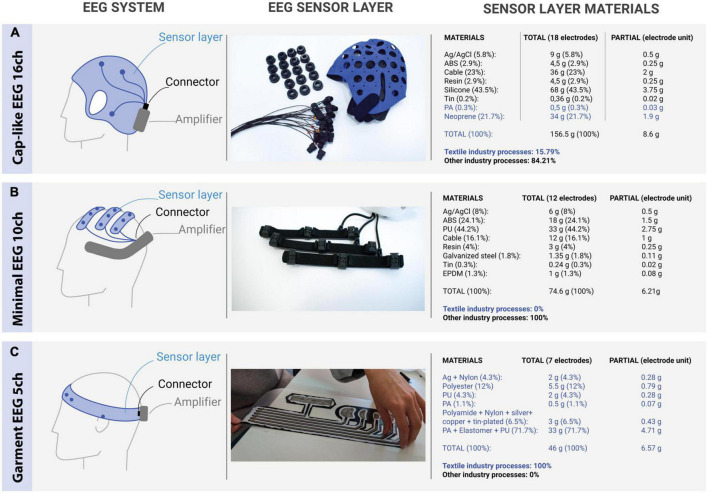
Different technologies for electroencephalogram (EEG) devices with specific focus on the EEG sensor layer. **(A)** Example of “shower-cap” research-oriented EEG systems. **(B)** Example of “minimalistic” application-oriented dry EEG systems. **(C)** Proof-of-concept “garment” EEG system. For a complete review of similar devices from different manufacturers, see Niso et al. (2023). In all the EEG devices, we differentiate between the EEG sensor layer, the connector, and the amplifier. In panels **(A,B)**, the sensor layer is implemented with plastics, metals, glues and materials from the electronic devices industries, while in panel **(C)**, “garment” sensor layers are implemented with materials and manufacturing processes from the textile industry only. The blue text in the right panel represents materials and manufacturing processes from the textile industry. ABS, acrylonitrile butadiene styrene; PA, polyamide; PU, polyurethane; EPDM, ethylene propylene diene monomer rubber.

Mobile EEG systems are composed by three elements: (1) the EEG sensor layer (i.e., EEG electrodes, signal transmission and head support); (2) the EEG amplifier (analogic, digital and communication electronics); and (3) the interface between them (connector), see Figure 1. The sensor layer is the component that contributes most to the usability/ergonomics of the whole system, and thus, is key for its acceptability in out-of-the-lab environments (Biasiucci et al., 2019; Casson, 2019; Niso et al., 2023). This layer, and specifically the EEG electrodes, traditionally rely on wet electrodes (Figure 1, first row), which require the application of an electrolytic substance between the sensor and the scalp. Although these electrodes are the gold-standard in medical applications, the need for such a substance reduces their usability and acceptance. Dry-EEG electrodes do not require the application of electrolytes (Figure 1, second row), which improves setup, cleaning time, ergonomics and comfort, especially for non-experts (Lopez-Gordo et al., 2014). Dry electrodes operate with higher contact impedances and thus are more prone to artifacts when compared to wet electrodes. However, this issue is partially compensated by the use of higher input impedance amplifiers (Shad et al., 2020). There has been a lot of research in new conductive materials and electrode shapes to create novel dry-EEG electrodes: different conductive metals and coatings (Lopez-Gordo et al., 2014), hydrogels (Li et al., 2021; Pei et al., 2022), polymers (Leleux et al., 2014), conductive inks (Ferrari et al., 2020), graphene (Ko et al., 2021), organic transistors (Venkatraman et al., 2018), conductive/smart textiles (Tseghai et al., 2020), among others.

Conductive or smart textiles offer significant advantages for the development of EEG sensors (usually referred to as textile-EEG or textrodes), given that they allow for complete integration of the EEG sensor layer into a garment (to differentiate between the two approaches, we will refer to the latter as “Garment-EEG”). These sensors or garments can be built by combining conductive and isolating fibers, making them light, soft and, bendable, which increases user-comfort and reduces fabrication cost (see right column in Figure 1 for a comparison of the use of different textile and non-textile materials for manufacturing different EEG devices). They can also inherit some relevant properties from the textile industry, like being breathable and washable. There are successful examples of biosignal electrodes based on textiles (Márquez et al., 2013) that measure ExG signals within the millivolt range, like electrocardiogram (ECG), electromyogram (EMG) and electrooculogram (EOG) (Catrysse et al., 2003; Acar et al., 2019). However, EEG is measured in the microvolt range, and obtaining an acceptable signal-to-noise ratio is far more challenging. Note that, while standard wet EEG has impedances below 5 KΩ, textile (and other dry) sensors are two orders of magnitude above (in the range of 100 s of KΩ), and thus, have higher levels of noise. To date, only a few research studies have proposed innovative EEG electrodes with different conductive textile materials, such as silver-plated or copper-plated fabrics (Tseghai et al., 2020). A recent review of the literature on textile EEG suggested that the lack of relevant progress in this field is mainly due to 2 factors. On the one hand, the new textile EEG systems are limited to publishable research, with no incremental or further innovative development. On the other hand, these published developments do not generally report quantitative comparisons with standard EEG systems to assess their value (Tseghai et al., 2020). When generalizing from the electrode to the entire sensor layer, all proposed textile EEG systems always rely on non-textile materials (metals or plastics) to complete the sensors, transmissions, or cap support. For instance, by developing textile electrodes that are used to contact standard metal electrodes (Fleury et al., 2017), or by connecting the textile electrodes via metal snaps to standard cables (Löfhede et al., 2012; Arnin et al., 2013).

In this paper, we move one step further in the state-of-the-art in textile EEG. We characterize the first EEG sensor layer implemented using only materials and manufacturing processes from the textile industry (Figure 1, third row). In other words, the complete sensor layer is a garment by design and manufacture. The contribution is the unified design of the EEG electrodes, the shielded transmission and the head support using conductive and isolating textile materials. As a first proof of concept evaluation of the technology, we ran an experimental validation, measuring EEG activity over the forehead area of healthy participants (sub-hairline EEG), as is usually done for the evaluation of novel EEG approaches (Leleux et al., 2014; Lepola et al., 2014; Bleichner and Debener, 2017; Venkatraman et al., 2018; Ferrari et al., 2020). The new EEG system (Garment-EEG) was quantitatively characterized with respect to a state-of-the-art Ag/AgCl dry EEG system (Dry-EEG) in terms of: (1) skin-electrode impedance; (2) EEG activity; (3) artifacts; and (4) user ergonomics and comfort.

## Materials and methods

To characterize the new EEG sensor layer, we designed two different headbands to monitor EEG from people’s forehead, at positions F7, Fp1, Fp2, and F8, according to the International 10/20 system. One of them was made exclusively with textile materials (Garment-EEG) and the other integrated standard metal electrodes and coaxial cables (Dry-EEG). This Dry-EEG technology was selected as baseline and state-of-the art for the comparison as it has already been benchmarked with respect to a medical-grade gel-EEG system in the context of electrophysiological measurements and brain-computer interfaces (Schwarz et al., 2020). Each of the two headbands contained four recording electrodes, as well as reference and ground electrodes. The headbands were equipped with identical connectors at the back, where the amplifier was attached. As this research constitutes the first-in-human validation of the technology, we opted for a simplified setup in which we measured sub-hairline EEG to facilitate access and replicability. This approach enables us to focus on the skin-electrode properties and quality of the electrophysiological data, while mitigating artifacts derived from contact with hairy areas (placement, difference type of hair, head morphology, etc.).

### Garment-EEG headband

**FIGURE 2:**
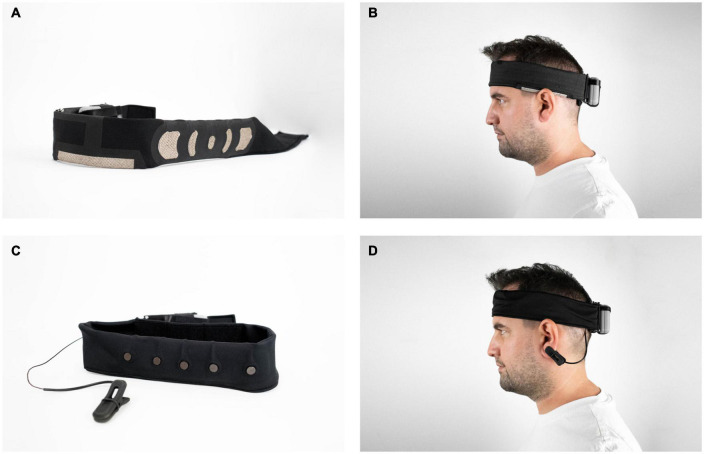
Garment-EEG and Dry-EEG headbands. **(A)** Detail of Garment-EEG electrodes shape and location. **(B)** Garment-EEG headband worn by one participant. **(C)** Detail of Dry-EEG electrodes shape and location. **(D)** Dry-EEG headband worn by one participant.

The novel Garment-EEG headband integrated sensors and signal transmission using only textile materials (Figures 2A, B). The textrodes were implemented using 3-strand silver-coated Nylon conductive yarns with a linear resistance of 114 Ω/m. They were embroidered in 3 directions (0°, −45°, and 45°) with a distance between yarns of 1 mm. Electrodes at Fp1 and Fp2 covered an area of 2.8 cm^2^, while F7 and F8 electrodes covered an area of 7.4 cm^2^, allowing appropriate electrode-skin contact for different head sizes. The ground electrode was placed on Fpz and covered an area of 1.5 cm^2^. The reference electrode had a long rectangular shape of 18 cm^2^ to facilitate the contact on the upper union between the left ear and the head regardless of the head perimeter.

By overlapping different textile layers, the signal transmission from the sensor to the connector was implemented mimicking a coaxial cable with active shielding. First, the transmission wire was embroidered with 8-strand silver-coated Nylon yarns, with a linear resistance of 15 Ω/m. This conductor was insulated using vinyl by heat-sealing at a temperature of 150°C for 10 s. Then, an additional conductive layer was added to shield both sides, using Ultraflex Tape Zell RS conductive fabric, made of PA/Nylon 6.6 coated with silver, copper, and tin, and with surface resistivity of less than 0.02 Ω. The final overlay, to isolate and fix the transmission to the fabric, was made with vinyl sealed at 150°C for 10 s.

The connection between the textrode and the transmission core was made by embroidering the former directly onto the latter. The connection between the transmission and the connector was made through a rigid PCB with copper pads sewn with conductive wire made of silver-coated polyamide/polyester with a linear resistance of less than 530 Ω/m. The support headband was made of polyester fabric with a satin structure and a grammage of 120 g/m^2^.

### Dry-EEG headband

The Dry-EEG system integrated Ag/AgCl electrodes, welded on coaxial cables, onto a similar headband (Figures 2C, D). The 4 recording electrodes were located at F7, Fp1, Fp2 and F8 positions. The ground electrode was placed on Fpz, and the reference electrode was on the left ear with an earclip. These electrodes had a circular shape with a diameter of 0.8 cm. The cables included a silver platted copper core (0.45 mm of diameter, resistance of 0.158 Ω/m), a PTFE tape insulation, a silver platted copper shielding layer, and an extruded FEP jacket. The connection between the coaxial cables and the connector was made by welding. The support headband was made of polyester fabric with a satin structure and a grammage of 120 g/m^2^ (similar to the textile one).

### EEG connector and amplifier

The two EEG headbands were equipped with the same high input impedance amplifier (50 GΩ) that had a CMRR of 100 dB. This helped reduce noise and minimize imbalances in skin-electrode impedance [which for dry-EEG, can be in the order of 100–200 KΩ, (see Lopez-Gordo et al., 2014; Shad et al., 2020)]. The system provides active shielding for the sensors and a DRL circuit to reduce noise. This amplifier samples the 4 EEG channels at 256 Hz and uses Bluetooth Low Energy (BLE) to transmit the data to a laptop. To connect the sensor layer and the amplifier, we used a commercial 20-position board-to-board connector with gold-plated phosphor bronze contacts and a liquid crystal polymer cover.

### Experimental validation

We carried out two experimental studies. In the first one, we characterized the impedance of the electrodes and signal transmission of the new Garment-EEG system, while in the second, we quantified common spontaneous (frequency domain) and evoked (time-frequency domain) EEG patterns of activity.

#### Participants

Six healthy volunteers participated in the first study (5 females, 1 male, age: 30.2 ± 5.8 years) and ten different healthy volunteers participated in the second study (5 females, 5 males, age: 27.8 ± 3.7 years). The participants were recruited from Bitbrain’s volunteer database and received an economic compensation of 25€. All of them signed an informed consent form and were debriefed about the study purpose and their rights regarding the physiological and demographic data collected from them.

#### Study 1: impedance analysis

We quantified impedances of the sensor layer (including the electrode and transmission) of both Garment-EEG and Dry-EEG systems. As baseline, we performed an additional measurement (Wet-EEG) in which the Dry-EEG headband was used with an electrolytic substance (SAC2 conductive gel, by Spes Medica) after cleaning the skin with abrasive gel (NeurPrep, by Spes Medica). This skin preparation with abrasive gel is a common procedure in clinical and research EEG applications. To simulate real-world conditions in the evaluation of the Garment-EEG and Dry-EEG impedances, the skin of the subjects was only cleansed with make-up remover wipes.

We used a PalmSens4 with a MUX8-R2 multiplexer (PalmSens, Houten, Netherlands) to measure the skin-electrode impedance as a function of logarithmically sampled frequency, ranging from 0.1 to 30 Hz with 10 sample points. We repeated the impedance measurements 3 times for each of the 4 electrodes.

#### Study 2: EEG activity analysis

In this study, participant’s brain activity was monitored using the two headbands sequentially. They performed the same tasks using both technologies in a cross-over manner, half of them starting with the Garment-EEG and the other half starting with the Dry-EEG [serial setup for EEG comparisons, (see Gargiulo et al., 2010; Kutafina et al., 2020)]. The tasks were:

• **Task 1**: 3 min of resting state with eyes closed.
• **Task 2**: 3 min of resting state with eyes open.
• **Task 3**: 80 cue-guided reaching movements with the right arm. Participants completed 4 blocks of 20 movements each. They faced a screen on which two different visual cues were displayed: “rest” (with a random duration of 7 ± 1 s) and “movement” (with a fixed duration of 5 s). They were instructed to blink and make any movement to adjust their position after the change from the “movement” period to the “rest” period.
• **Task 4**: artifact induction, where the following actions were executed for 10 s each, to contaminate the EEG signals: (i) the experimenter moves both hands above the participant’s head; (ii) tongue movements; (iii) jaw movements; (iv) blinking; (v) lateral and vertical eye movement; (vi) vertical head movement; (vii) horizontal head movement; (viii) up and down shoulder movement; (ix) up and down movement with both arms.

The raw EEG data recorded in this study is available as Supplementary material.

### EEG analysis

The EEG signals recorded with both technologies were quantified and compared using a frequency analysis (tasks 1, 2, and 4) and a time-frequency analysis (task 3).

#### Frequency analysis of EEG during resting state/artifact induction (tasks 1, 2, and 4)

The eyes-closed and eyes-open resting state (Tasks 1 and 2), and the artifact induction (Task 4) EEG recordings were analyzed in terms of their power spectral density.

##### Preprocessing

First, we removed outlier amplitude values from each EEG recording. For that, we high-pass filtered the signals at 60 Hz, with a 4-th order non-causal Butterworth filter, applied a Hilbert transform, and z-scored the values. Those samples going beyond 5 standard deviations above the mean were marked as outliers and discarded in the original signal, filling the gaps by linear interpolation. This step was not applied to the signals of the artifact induction task, since in that case, the objective was to compare the occurrence of artifacts in both technologies.

##### Processing

We filtered the EEG signals between 0.1 and 100 Hz, using a 4-th order non-causal Butterworth filter. Subsequently, we estimated their power distribution between 0 and 60 Hz using a fast Fourier transform (FFT) with a 1 s hamming window, zero-padded to 1024 points (0.25 Hz resolution) and 30 ms of overlap, and computed the logarithm of the obtained power values.

##### Statistics

For the statistical comparisons, we averaged the power values of the four electrodes. For this *average* electrode, we computed the mean power for each participant and headband in five frequency bands: 0–3 Hz (delta), 4–7 Hz (theta), 8–12 Hz (alpha), 13–30 Hz (beta), and 45–55 Hz (electrical noise). These ranges cover the most common frequency bands in EEG analyses, as well as the influence of electrical contamination, which can been used as a surrogate of electrode impedance (Insausti-Delgado et al., 2021). A paired Wilcoxon signed-rank test was used to test for statistical differences between both headbands in each frequency band.

#### Time-frequency analysis of EEG during movement execution (task 3)

The EEG data recorded during the execution of reaching movements was analyzed in the time-frequency domain to quantify the event-related desynchronization/synchronization (ERD/ERS) of sensorimotor rhythms (Pfurtscheller and Lopes da Silva, 1999).

##### Preprocessing

First, we removed trials that contained unusually high amplitudes. Trials showing amplitude values higher than 250 μV in any of the four electrodes were marked as artifacts and discarded.

##### Processing

We filtered the EEG signals between 0.3 and 30 Hz, using a 4-th order non-causal Butterworth filter. Subsequently, we computed the time-frequency representation of all the trials using Morlet Wavelets in the frequency range 5–30 Hz, with a frequency resolution of 0.25 Hz. The ERD/ERS maps were computed as the percentage of power decrease/increase with respect to a resting state baseline ([−5, −2] s).

##### Statistics

To compare the ERD/ERS obtained with the Garment-EEG and the Dry-EEG headbands, we first averaged the time-frequency representation of all the trials of each subject, and then averaged across electrodes, obtaining a time-frequency matrix per subject. Subsequently, for each time-frequency bin, we applied a paired Wilcoxon signed-rank test. No correction for multiple comparisons was used.

### Comfort and perception assessment

After completion of the EEG recordings with each sensor layer, participants filled a comfort and perception questionnaire, in which they were asked about the following four points:

• Global comfort of the system, including the electrodes, the headband and the amplifier, on a scale from 1 (“Not comfortable at all”) to 7 (“Very comfortable”).
• Comfort of the EEG electrodes and their contact with the forehead skin, on a scale from 1 (“Not comfortable at all”) to 7 (“Very comfortable”).
• Weight perception of the system, using the following scale: 1 “I do not feel the weight”, 2 “Noticeable but not bothersome,” 3 “Noticeable but little bothersome,” and 4 “Very noticeable and bothersome.”
• Perception of stability of the system, using the following scale: 1 “I feel it stable,” 2 “I feel it a bit unstable,” 3 “I feel it unstable.”

## Results

### Impedance analysis

**FIGURE 3:**
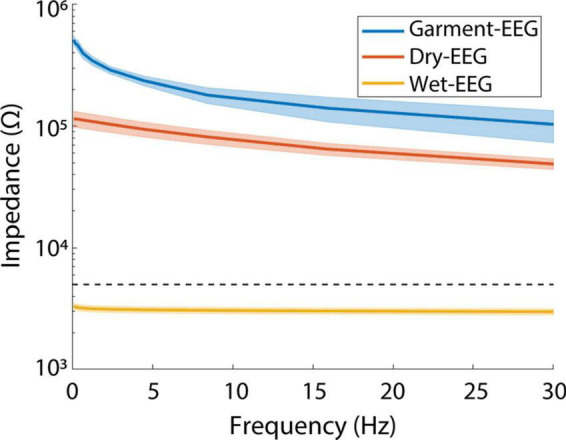
Impedance results for Garment-EEG, Dry-EEG, and Wet-EEG. Impedance is displayed as a function of the frequency. The values for all the subjects, electrodes and repeated measurements for each electrode are averaged for each technology. The shades represent the standard error of the mean. Notice that the *y*-axis is displayed in a logarithmic scale. The dashed horizontal line displays the value of 5 KΩ, as standard threshold for good impedance in wet EEG systems (Nuwer et al., 1998).

Figure 3 shows the impedance values as a function of the frequency for the Garment-EEG, Dry-EEG and Wet-EEG (Dry-EEG using electrolytic gel). The average impedances at 1 and 10 Hz for the Garment-EEG were 368 and 171 KΩ, for the Dry-EEG were 110 and 78 KΩ, and for the Wet-EEG were 3.2 and 3.05 KΩ (below the gold-standard threshold of 5 KΩ). Notice that Garment-EEG has systematically higher impedances than Dry-EEG (around two-three times more), and both of them are two orders of magnitude above Wet-EEG. These results are in line with state-of-the-art values reported for wet and dry EEG systems (Lopez-Gordo et al., 2014; Shad et al., 2020).

### Frequency analysis of EEG during resting state/artifact induction

**FIGURE 4:**
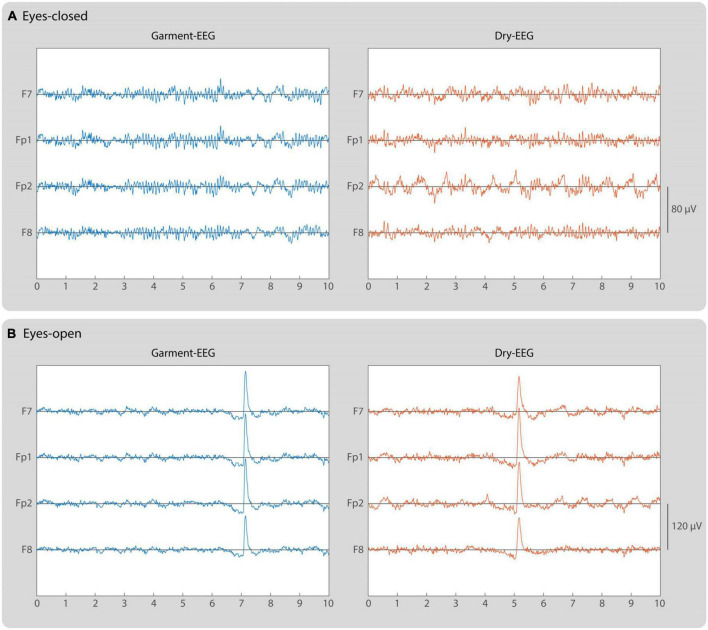
Electroencephalogram signals recorded with Garment-EEG and Dry-EEG technologies. Time series representing 10-s EEG traces of the 4 electrodes recorded with each system during **(A)** eyes-closed and **(B)** eyes open. Left panels correspond to the measurements with the Garment-EEG, while right panels correspond to the Dry-EEG. The signals are bandpass filtered between 0.5 and 30 Hz. Notice that the vertical scale is different for eyes-closed and eyes-open signals.

**FIGURE 5:**
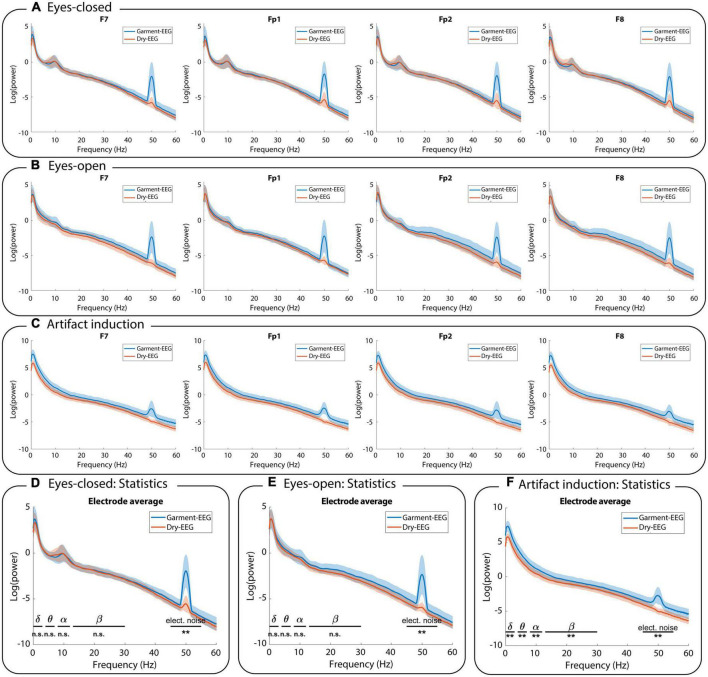
Frequency analysis. Power spectral density comparison between the EEG activity measured with the Garment-EEG and Dry-EEG during **(A)** eyes-closed, **(B)** eyes-open, and **(C)** artifact induction. In panels **(A–C)**, each subplot displays one of the four forehead electrodes (F7, Fp1, Fp2, and F8). Statistical comparisons between both headbands on five frequency bands during **(D)** eyes-closed, **(E)** eyes-open, and **(F)** artifact induction. n.s., non-significant; **p < 0.01.

Both the Garment-EEG and the Dry-EEG system provided similar EEG signals in terms of morphology and amplitude during the resting state measurements (Figure 4). Strong alpha waves are visible during eyes-closed, which are then suppressed during eyes-open, where eye blinks become noticeable. Figure 5 displays the power spectral density analysis of the EEG signals recorded with both headbands during eyes-closed resting state (Figure 5A), eyes-open resting state (Figure 5B), and during the generation of artifacts (Figure 5C). Overall, the signals measured during resting state with the Garment-EEG and the Dry-EEG headbands displayed equivalent power spectral density values at the frequencies usually analyzed in EEG studies (i.e., below 30 Hz). During eyes-closed resting state, a clear alpha peak at ∼10 Hz can be observed in frontopolar (Fp1 and F2) and frontal (F7 and F8) locations using both technologies (Figure 5A). This alpha activity is not so evident during eyes-open (Figure 5B). Noticeably, the Garment-EEG headband was found to be more susceptible to artifacts. This can be seen as higher power at 50 Hz in all the channels (Figures 5A, B), as well as higher broadband power during the artifact induction task (Figure 5C). For both resting state measurements, the EEG power in delta, theta, alpha and beta frequencies was not significantly different between the Garment-EEG and the Dry-EEG (Figures 5D–E; *p* > 0.05 in all cases). Conversely, during the induction of artifacts EEG, the power in these frequencies was significantly higher for the Garment-EEG headband than for the Dry-EEG headband (Figure 5F; *p* < 0.01 in all cases). For the 45–55 Hz frequency band, the power was significantly higher with the Garment-EEG headband both during resting state and during artifact induction (Figures 5D–F; *p* < 0.01 in all cases).

In a subsequent analysis, we investigated which of the actions of the artifact induction task contributed most to the differences between both technologies. We repeated the statistical comparisons on the power values obtained from the 10-second segments of signal corresponding to each action (see Supplementary Figure 1). The actions involving larger motions, such as the experimenter moving their hands above the participant’s head, as well as vertical head movements and arm movements, produced significant differences in multiple frequency bands between the technologies, whereas tongue and jaw movements produced significant differences in delta only. For four of the actions (eye blinks, eye movements, horizontal head movement, shoulder movement), there were no significant differences between the two technologies in either of the delta, theta, alpha, and beta frequencies.

### Time-frequency analysis of EEG during movement execution

**FIGURE 6:**
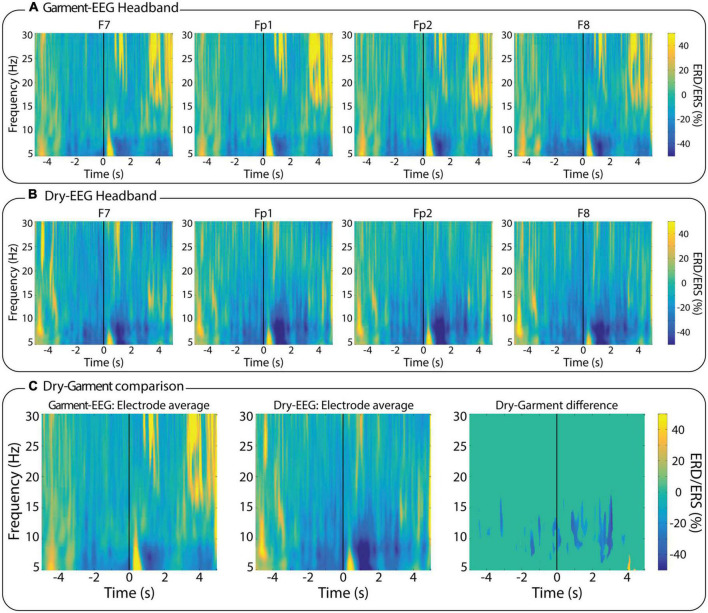
Time-frequency analysis. Event-related (de)synchronization (ERD/ERS) maps of the EEG activity measured during the reaching movements with **(A)** the Garment-EEG headband, and **(B)** the Dry-EEG headband. Each subplot in panels **(A,B)** displays one of the four forehead electrodes (F7, Fp1, Fp2, and F8). **(C)** Comparison between the two technologies; (left) average of all the Garment-EEG electrodes, (center) average of all the Dry-EEG electrodes, (right) pairwise statistical comparison between both headbands.

Figure 6 shows the ERD/ERS maps of the four electrodes with the Garment-EEG headband (Figure 6A) and with the Dry-EEG headband (Figure 6B). Given the distant location of the four electrodes with respect to the motor cortex, which is where this motor-related activity originates, the ERD of sensorimotor rhythms is of low magnitude with both technologies. When directly comparing the ERD magnitude of the *average* electrode of both systems, the Dry-EEG headband produces a stronger ERD (Figure 6C, left and center panels). The time-frequency bins that showed a significant difference between both headbands (*p* < 0.05) were used to create a mask that was subsequently applied to the difference between the ERD/S map of the Dry-EEG headband and the ERD/S map of the Garment-EEG headband (Figure 6C, right panel). This analysis revealed that the Dry-EEG headband provided a significantly stronger ERD between 5 and 15 Hz during the execution of the reaching movements.

### Comfort metrics

**FIGURE 7:**
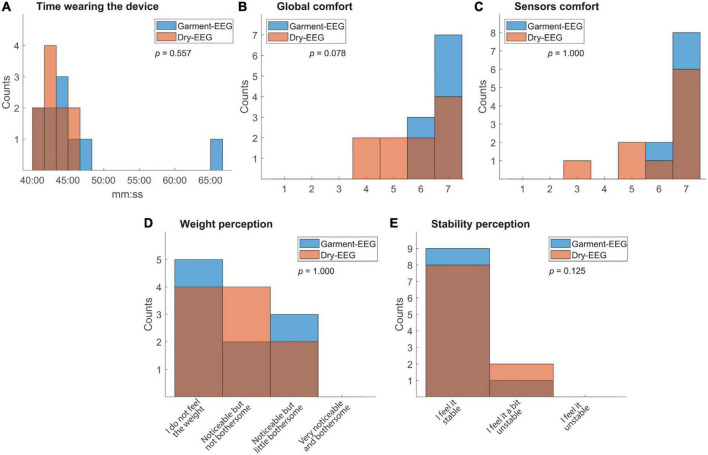
Comfort metrics. Histograms representing **(A)** the total time the subjects wore the headband, and subjective metrics, such as: **(B)** global comfort of the headband, **(C)** comfort of the sensors, **(D)** perception of the systems’ weight and **(E)** perception of the system’s stability. The *p*-values under the legend box represent the result of the paired Wilcoxon signed-rank test between the two distributions.

The time the participants spent with each headband ranged between 42 and 48 min, except for one case (Participant 1, session with Garment-EEG headband) where, due to technical problems, the session lasted 66 min (Figure 7A). The degree of comfort reported by the participants, both for the whole system and for the sensors part, was always high for the Garment-EEG headband and medium-to-high for the Dry-EEG headband (Figures 7B, C). The perception of the weight was distributed between “I do not feel the weight” and “Noticeable but little bothersome,” and the participants never reported any of the headbands to be bothersome (Figure 7D). The perception of stability was also positive, with most of the responses being “I feel it stable,” and none reporting the systems to be unstable (Figure 7E). Statistical comparisons were carried out for the five analyses using the Wilcoxon signed-rank test, but none of them reached the significance level of *p* < 0.05 (see *p*-values in Figure 7).

## Discussion

Accessible electroencephalographic (EEG) systems are the first step toward democratizing neurotechnology. Market indicators for wearables predict that in the next one or two decades, wearable neurotechnology will be widely adopted, much like other wearables several years ago (Johnson and Picard, 2020). Recent hardware innovations in dry EEG technologies have widened the portfolio of tools available for monitoring the brain. However, despite the progress seen over the last decade, we cannot yet consider that dry electrodes have completely revolutionized the way EEG is used.

User acceptance in terms of ergonomics, usability and price are the main obstacles for EEG systems to establish neurotechnology and non-invasive brain-computer interfaces as common technology. In principle, EEG sensor layers built with textiles and encapsulated in garments have potential to enable neurotechnology that is naturally accepted by people in their daily lives. In addition to this, EEG implementation in the textile industry would lead to lower manufacturing costs and much less pollution when compared to metal and plastic industries. In this paper, we propose and quantitatively characterize a proof of concept for an EEG measuring garment; a system that uses, exclusively, textile materials for its sensor layer. The evolution and optimization of this technology might open the door to its application out of scientific laboratories or clinics, either for real-world research, for home-based wellness applications, or for patient diagnostics, treatment, and follow-up.

### Signal quality and artifacts

Overall, the proof-of-concept Garment-EEG proposed in this study provided EEG recordings that were comparable to those of state-of-the-art, metal-based dry EEG electrodes. In terms of resting state recordings with eyes open and eyes closed, the signals in the frequency domain had equivalent power distributions for both technologies, and there were no significant differences in power between the Garment-EEG and the Dry-EEG at frequencies below 30 Hz (i.e., the ranges usually analyzed in EEG applications). This includes the alpha waves visible in the eyes closed condition and its blocking when eyes are open (Klimesch, 1999).

The motor-related evoked activity produced by arm movements, measured as time-frequency ERD/ERS maps, was visible with both systems over the measured frontal areas, but with a considerably lower magnitude than what is normally measured at the motor cortex during this type of tasks (López-Larraz et al., 2014). Dry-EEG provided slightly stronger ERD maps than Garment-EEG. Although these results might not be conclusive, there are two possible causes: (1) it could be that artifacts produced during the execution of upper limb movements (Bibián et al., 2022) affect the recording with textile electrodes more than with dry electrodes; and (2) the reference of the Garment-EEG is in the upper part of the ear, being closer to the sensory-motor area than in the Dry-EEG, which could lead to an attenuation of the ERD.

Garment-EEG was more prone to getting affected by artifacts, which was seen as significantly higher 50 Hz contamination and significantly higher broadband power during the artifact induction task. Artifacts are one of the most limiting problems in EEG research (Urigüen and Garcia-Zapirain, 2015; López-Larraz et al., 2018), and therefore, the higher likelihood of Garment-EEG getting contaminated should not be disregarded. However, having more frequent artifacts does not invalidate the relevance of the technology. Artifact removal is a standard procedure (and even a necessary step) in most clinical and research EEG applications (Urigüen and Garcia-Zapirain, 2015), and recent research is currently targeting specific techniques for low density EEG (Lopez et al., 2022). Therefore, while there should be lines of work trying to minimize the appearance and impact of artifacts in this new technology, there can also be progress in the field under the assumption that this type of technology can overcome this limitation with its ability to effortlessly acquire massive amounts of data and exploit advanced machine learning techniques (Craik et al., 2019). The fact that signals were comparable in less artifacted conditions, such as resting state recordings, is an encouraging result that supports further research and development to improve this technology.

Actions that involved large movements, and especially the experimenter moving their hands above the participant’s head, caused the largest artifacts in the Garment-EEG recordings. The reason behind this might be that these movements change the capacitive coupling of the sensors with the mains, as described in Metting Van Rijn et al. (1990), affecting the impedance of the electrodes and transmission lines. Poor impedance is one of the primary factors contributing to the presence of artifacts, and could be the source of the differential impact of artifacts on the Garment-EEG as compared to the Dry-EEG. Impedances of Dry-EEG electrodes have been reported to be in the range of 100–200 KΩ over non-hairy areas (Lopez-Gordo et al., 2014; Shad et al., 2020). This is the reason why high input impedance amplifiers, like the one used in this study, are required (preferably in the order of GΩ) (Niso et al., 2023). As can be seen in our study, the impedances of the Garment-EEG and the Dry-EEG systems were consistent with those values, although they were around two to three times higher with the textile-based system. Conductive textiles cannot yet offer resistance values at the level of metals, and the fact that both the electrode and the transmission line of the Garment-EEG were made with textiles justifies these higher impedance values. Strategies to optimize the electrode-skin contact (Soroudi et al., 2019; Song et al., 2020) or to maximize the efficacy of the transmission lines (Leśnikowski, 2011) might be a reasonable starting point to continue optimizing this technology.

### Applications of garments to measure EEG

The use of smart textiles to manufacture EEG devices is a growing trend with the potential of large end-user acceptance and lower costs. One of the main critiques pointed out by a recent review in Textile-EEG is that most of the systems developed in the last few years are not further developed and remain only as published papers, without a clear progress toward usable and marketable devices (Tseghai et al., 2020). The authors also evidenced a lack of publications with quantitative analysis and from industrial research, which slows the progress in the field (Tseghai et al., 2020). We believe that public-private investment is key to foster the development of radically new technologies such as the one proposed in this study. Once this type of technology reaches an appropriate level of maturity, there is a vast range of applications that could easily benefit from having EEG devices integrated as garments. These could be divided into three contexts: medical, research and wellness/leisure.

While the use of neurotechnology to allow brain control of devices out of the laboratory (e.g., videogames, domotics or even rehabilitation) might still require several years of research and innovation to become a reality, ambulatory brain monitoring is a promising near-term medical application, for instance, for patient follow-up, seizure detection or sleep assessment (Cannard et al., 2020; Tseghai et al., 2020). In sleep studies, it could be used as a relatively cheap triage tool for patients that require a full polysomnographic study (Rundo and Downey, 2019). Epileptic patients can also require long term monitoring of the brain during daily life activities, and it could be easily done with a wearable textile EEG device (Hasan and Tatum, 2021). For the monitorization of newborns, or for patients in need of intensive care, brain monitoring with soft systems like textiles would also constitute a clear advantage (Löfhede et al., 2012). Sub-hairline and low density EEG montages have been described and implemented as part of the clinical toolkit when a fast and easy EEG is required or when EEG technicians are not available (Young et al., 2009; Tanner et al., 2014; Brenner et al., 2015; Touchard et al., 2021). These types of montages maintain a high specificity and are often a go-to for emergency situations.

In the mid-to-long term, this new technology in combination with modern artificial intelligence techniques, could be used to discover and track biomarkers related with the progress or treatment of neurological disorders, such as cognitive decline (Huggins et al., 2021), Parkinson’s disease (Scholten et al., 2020), stroke (Ray et al., 2020; Wilkinson et al., 2020) or spinal cord injury (López-Larraz et al., 2015). In addition to monitoring applications, these systems could also be integrated for home-based therapies, such as for motor rehabilitation (Bundy et al., 2017; Escolano et al., 2020), cognitive enhancement (Escolano et al., 2014), or closed-loop neurostimulation during sleep (Lee et al., 2020; Esparza-Iaizzo et al., 2021).

In terms of research applications, the fields that could most clearly benefit from this type of system are those that involve out-of-the-lab brain monitoring. Inexpensive and easy to wear EEG devices would enable large-scale recordings in ecological conditions (Dikker et al., 2017, 2021). This might constitute a breakthrough in the way we study the human brain (Matusz et al., 2019). On the one hand, brain activity could be measured in contexts where it is currently not possible, such as during daily life activities. On the other hand, the resources currently required to execute a scientific study with few dozens of participants, might allow to record hundreds or thousands of them.

In addition to clinical and research applications, brain monitoring with garments could be easily adopted for neuroscience applications in education, wellness, sports or industrial environments, incorporating these measurements to the range of bio-signals that are currently acquired by smart watches, rings, or chest-bands (Peake et al., 2018; Cannard et al., 2020; Di Pasquale et al., 2022).

### Limitations

As this work represents the first proof-of-concept of Garment-EEG technology, there are several limitations that should motivate future lines of work.

We chose a sub-hairline montage (i.e., forehead EEG) to assess the validity of the proposed technology and to compare it with dry EEG sensors. While this is a common approach for validating novel textile EEG electrodes (Löfhede et al., 2012; Arnin et al., 2013; Fleury et al., 2017), or other novel EEG sensing approaches (Leleux et al., 2014; Lepola et al., 2014; Bleichner and Debener, 2017; Venkatraman et al., 2018; Ferrari et al., 2020), many EEG applications require the placement of electrodes over areas of the head with hair. Therefore, further research should explore the possibilities of improving textile EEG technology to measure over areas of the head with hair. For instance, this could be achieved by developing electrodes that can penetrate though hair or by using electrolytic substances in combination with the textrodes.

The comparison between the Garment-EEG system and the Dry-EEG system was made in terms of electrode impedance, resting state activity, modulation of sensorimotor oscillations, and robustness against artifacts. Other types of EEG patterns of activity should be studied in future research, such as event-related potentials (Luck, 2014), which will require investigating different protocols such as P300, steady-state evoked potentials, or error-related potentials, among others. Additionally, note that we followed a “serial” method for the EEG comparison, meaning that the Garment-EEG and the Dry-EEG were positioned with a “same place different time” configuration. A different approach would be to use a “parallel” method, or “same time different place” (Kutafina et al., 2020). This second method will allow for performing additional quantitative analyses of the time series recorded with both technologies.

Among the measured tasks, one involving motor execution for measuring ERD/ERS activity was included. The results showed that neither the Garment-EEG nor the Dry-EEG devices provided accurate measurements of ERD/ERS activity, as the electrodes were not placed close to motor areas. This might make the analysis less informative, although future research could exploit advanced machine learning methods to predict activity from central brain areas using sensor placed on distant locations (Fickling et al., 2020).

Although the two headbands were designed as similarly as possible to facilitate their comparison, there are two differences in their design that might have influenced the obtained results. The first difference was electrode size. Anticipating that textile electrodes would have higher contact impedances than metal electrodes (due to the lower conductive properties of conductive threads vs Ag/AgCl alloys), we designed them with a larger size to increase their contact area, which lowers their impedance. To ensure proper contact with different head sizes, textrodes were made in varying sizes depending on their location. Adult head circumference statistics were taken into consideration to guarantee that the electrodes would provide proper contact for head sizes ranging from the 5th percentile of female heads to the 95th percentile of male heads. The second difference was the location of the reference electrode. The Dry-EEG system included an earclip to be placed in contact with the left earlobe, as commonly done in EEG wearable devices (Niso et al., 2023). Implementing an equivalent contact point in the earlobe using only textrodes and textile transmission would be challenging. Therefore, we opted to use the contact point of the headband with the upper part of the ear. Note that both locations can be valid for referencing EEG measurements and, in most cases, they will provide almost equivalent results (Nunez and Srinivasan, 2006). However, an electrode placed on the upper part of the ear might capture more electrical activity originating in the brain than an earlobe electrode. Also, the contact impedance might be slightly different for both locations, causing differences in how artifacts affect the signals. Both factors can potentially affect signal morphology. Future lines of work should focus on investigating the relevance of parameters such as the size of the textrodes and the location of the reference on the obtained measurements.

## Conclusion

This paper presented the first proof of concept of a garment to measure EEG activity. We compared the Garment-EEG with a state-of-the-art Ag/AgCl dry EEG system in terms of spontaneous and evoked EEG activity, artifacts, skin-electrode impedance, and comfort. Under favorable recording conditions (low level of artifacts), the Garment-EEG provided comparable measurements to the metal-based EEG system, although it is more prone to artifacts in adverse conditions, due to poorer contact impedances.

One of the most relevant advantages of this innovation is that it opens the door to creating EEG technology that can be broadly adopted by the general population, due to its comfort and its reduced manufacturing cost. This could allow large scale recordings for clinical and non-clinical purposes in ecological conditions. To promote open science and replicability, we are providing public access to our datasets, thus releasing the first open-access dataset of an EEG sensor layer built exclusively with textiles and allowing its comparison with a metal-based dry EEG.

An important final remark about the opportunities that this technology could enable is that this paradigm shift in brain monitoring should not take place without paying sufficient attention to its inherent risks. Experts in neuroethics are already working on creating recommendations for researchers, manufacturers and regulators to facilitate a responsible development of the neurotech field and its safe integration in our daily lives (Yuste et al., 2017).

## Data availability statement

The original contributions presented in this study are included in the article/Supplementary material, further inquiries can be directed to the corresponding author.

## Ethics statement

Ethical review and approval was not required for the study on human participants in accordance with the local legislation and institutional requirements. The patients/participants provided their written informed consent to participate in this study. Written informed consent was obtained from the individual(s) for the publication of any potentially identifiable images or data included in this article.

## Author contributions

EL-L and JM conceived and designed the study. EL-L, CE, AR-M, LM, and AA prepared the technology and the experimental setup. EL-L and AR-M participated in the data collection. EL-L and CE analyzed the data with inputs from AR-M, LM, AA, and JM. EL-L and JM drafted the manuscript with inputs from CE, AR-M, LM, and AA. All authors revised and approved the manuscript.
