# Supplementary material for: A garment that measures brain activity: proof of concept of an EEG sensor layer fully implemented with smart textiles
Source: Front Hum Neurosci. 2023 May 26;17:1135153. doi: 10.3389/fnhum.2023.1135153 (PMC10250743; doi:10.3389/fnhum.2023.1135153)
Supplement: Supplementary file 11 [file Data_Sheet_11.docx]

Supplementary Material

# Dataset

## Dataset description

The complete description of the participants, equipment and experimental paradigm are included in the main text of the manuscript. This document provides the information to load the data and replicate or conduct further analyses to the ones presented in this paper.

## Participants and Set-up

Ten healthy individuals participated in this study. Their brain activity was monitored using the Garment-EEG headband and the Dry-EEG headband. The participants performed the same tasks using both systems in a cross-over manner, half of them starting with the Garment-EEG headband, and the other half with the Dry-EEG headband.

The Garment-EEG system is a novel EEG technology whose sensor layer (i.e., electrodes and transmission lines) are built using textile materials only. The Dry-EEG system is a headband that integrates standard Ag/AgCl electrodes and cables, to serve as comparison of the state of the art in dry EEG.

## Experimental protocol

The participants executed the following tasks:

- **Task 1 (EyesClosed)**: 3 minutes of resting state with eyes closed.
- **Task 2 (EyesOpen)**: 3 minutes of resting state with eyes open.
- **Task 3 (Movement)**: 80 cue-guided reaching movements with the right arm. Participants completed 4 blocks of 20 movements each. They faced a screen on which two different visual cues were displayed: ‘rest’ (with a random duration of 7±1 s) and ‘movement’ (with a fixed duration of 5 s). They were instructed to blink and make any movement to adjust their position after the change from the ‘movement’ period to the ‘rest’ period.
- **Task 4 (Artifacts)**: induction of artifacts, where the following actions were executed for 10 seconds each, to contaminate the EEG signals: *i)* the experimenter moves both hands above the participant’s head; *ii)* tongue movements; *iii)* jaw movements; *iv)* blinking; *v)* lateral and vertical eye movement; *vi)* vertical head movement; *vii)* horizontal head movement; *viii)* up and down shoulder movement; *ix)* up and down movement with both arms.

## Structure of folders

EEG files have been stored in separate folders for each study participant (Subject101 – Subject205). The folder of each participant includes separate folders for each task-headband combination. The name of each of these folders starts with a 2-digit code and the name of the task (‘01_EyesClosed’; ‘02_EyesOpen’; ‘03_Movement’; ‘04_Artifacts’), followed by the name of the technology used (‘Garment-EEG’ or ‘Dry-EEG’). Within each of these folders there is only one file, whose name represents the subject, the task, and the type of headband. The data is stored as .mat files, which should be opened in Matlab/Octave.

Participants that executed the experiment starting with the Garment-EEG, followed by the Dry-EEG, are coded as 101-105, while participants that executed the experiment starting with the Dry-EEG, followed by the Garment-EEG, are coded as 201-205.

## Structure of files

This is the structure of each of the .mat files:

- **Task 1 (EyesClosed)**: each file contains 3 variables.
  - EEG: electrodes×samples matrix, with 4 rows (number of electrodes) and 46.080 columns (number of samples of the 3-minute recording).
  - SR: sampling rate of the recording (256 in all cases).
  - Labels: name and order of the electrodes included in the ‘EEG’ matrix.
- **Task 2 (EyesOpen)**: each file contains 3 variables.
  - EEG: electrodes×samples matrix, with 4 rows (number of electrodes) and 46.080 columns (number of samples of the 3-minute recording).
  - SR: sampling rate of the recording (256 in all cases).
  - Labels: name and order of the electrodes included in the ‘EEG’ matrix.
- **Task 3 (Movement)**: each file contains 6 variables.
  - Block1, Block2, Block3, Block4: structures storing the data corresponding to the four 20-trial recordings. Each Block structure contains:
    - EEG: electrodes×samples matrix, with 4 rows (number of electrodes) and a variable number of columns (note that the duration of some intervals was random, and therefore the recordings did not have a fixed duration).
    - Events: vector with the 20 time instants corresponding to the moments where the participants were requested to start the movement. Before/after each of these events, there should be a resting period of 7±1 s, and a movement period of 5 s.
  - SR: sampling rate of the recording (256 in all cases).
  - Labels: name and order of the electrodes included in the ‘EEG’ matrix.
- **Task 4 (Artifacts)**: each file contains 3 variables.
  - EEG: electrodes×samples matrix, with 4 rows (number of electrodes) and a variable number of columns (note that the duration of this task was not fixed, although it is always close to 1.5 minutes).
  - SR: sampling rate of the recording (256 in all cases).
  - Labels: name and order of the electrodes included in the ‘EEG’ matrix.

## EEG data

Note that the EEG datasets are shared in raw format. No processing, filtering or artifact removal has been applied to the signals. To obtain equivalent results to the ones presented in the paper, see its “EEG analysis” section and apply the same *Preprocessing* and *Processing* steps.

# Supplementary Figures


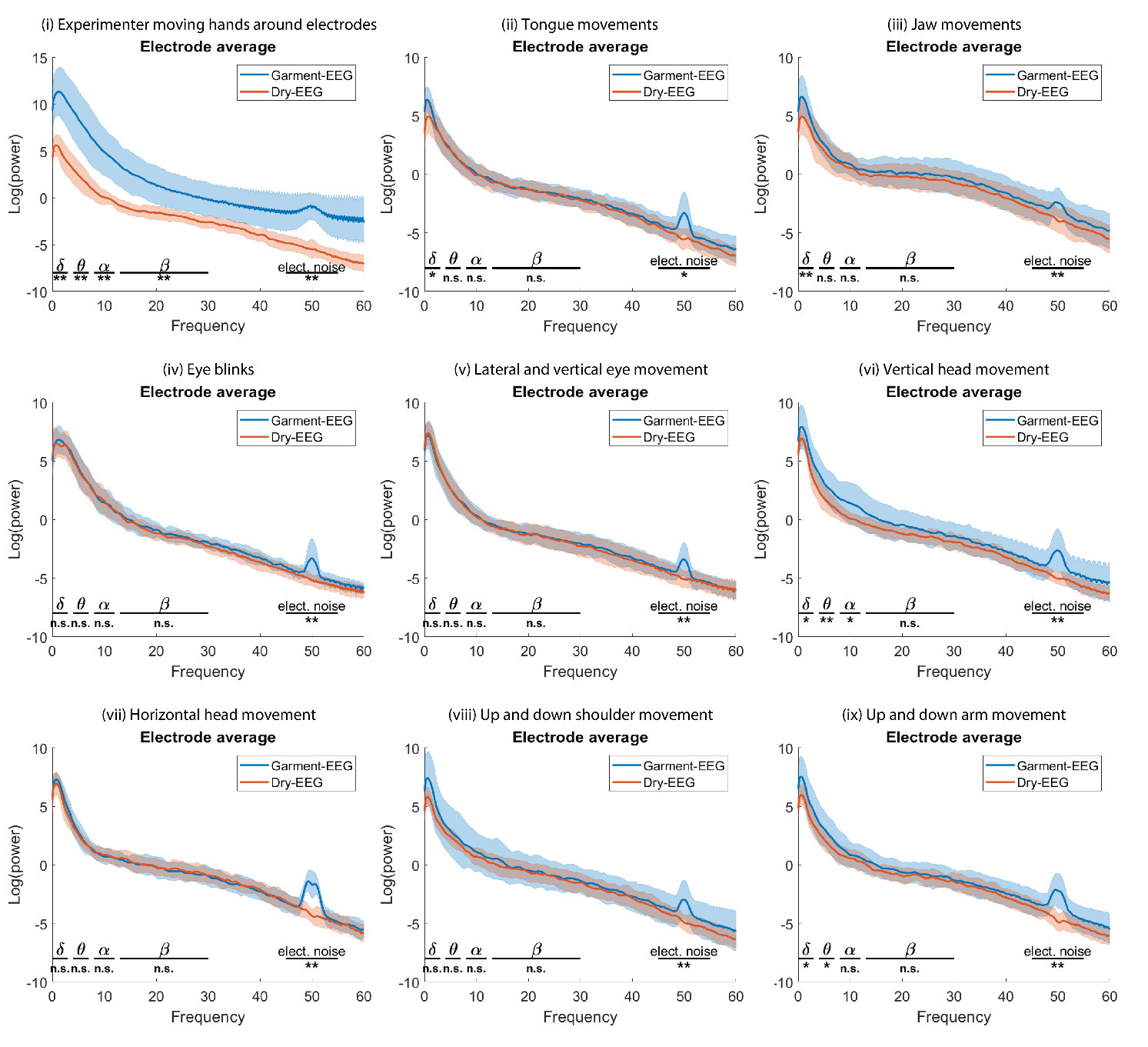


*Supplementary Figure 1. Frequency analysis of the artifact induction condition, separated by artifact type. Power spectral density comparison between the EEG activity measured with the Garment-EEG and Dry-EEG during different contaminating actions. Statistical comparisons are shown between both headbands on the five studied frequency bands. n.s. = non-significant; * p<0.05; ** p<0.01.*
